# The Anti-Aggregative Potential of Resolvin E1 on Human Platelets

**DOI:** 10.3390/molecules28145323

**Published:** 2023-07-11

**Authors:** Patrycja Szymańska, Bogusława Luzak, Katarzyna Miłowska, Jacek Golański

**Affiliations:** 1 Department of Haemostasis and Haemostatic Disorders, Chair of Biomedical Sciences, Medical University of Lodz, Mazowiecka 6/8, 92-215 Lodz, Poland; boguslawa.luzak@umed.lodz.pl (B.L.); jacek.golanski@umed.lodz.pl (J.G.); 2 Department of General Biophysics, Faculty of Biology and Environmental Protection, University of Lodz, Pomorska 141/143, 90-236 Lodz, Poland; katarzyna.milowska@biol.uni.lodz.pl

**Keywords:** platelets, aggregation, reactivity, anti-aggregative, collagen, GPVI, platelet membrane fluidity, omega-3 PUFAs, EPA, resolvin E1

## Abstract

Resolvin E1 is a metabolite of eicosapentaenoic acid (EPA) which is one of the omega-3 polyunsaturated fatty acids (omega-3 PUFAs). The antiplatelet properties of omega-3 PUFAs are well known, but the effect of resolvin E1 on platelets via the collagen receptors is extremely poorly reported. We investigated the effect of resolvin E1 on collagen-induced platelet aggregation, activation, and reactivity, and also platelet membrane fluidity. The ultimate and statistically significant results showed that resolvin E1 may inhibit platelet reactivity due to the reduction of collagen-induced platelet aggregation in platelet-rich plasma and isolated platelets, but not in whole blood. Also, resolvin E1 significantly reduced P-selectin exposure on collagen-stimulated platelets. Moreover, we demonstrated that resolvin E1 can maintain platelet membrane structure (without increasing membrane fluidity). The association between platelet reactivity and membrane fluidity, including resolvin E1 and collagen receptors requires further research. However, the goal of this study was to shed light on the molecular mechanisms behind the anti-aggregative effects of resolvin E1 on platelets, which are still not fully clarified. We also indicate an innovative research direction focused on further analysis and then use of omega-3 PUFAs metabolites as antiplatelet compounds for future applications in the treatment and prevention of cardiovascular diseases.

## 1. Introduction

Platelets are small, nucleus-free blood morphotic elements formed in the bone marrow from megakaryocytes and can live for up to 10 days. In a healthy adult, their physiological concentration in the blood is 150–400 × 10^9^/L [1]. Platelets prevent bleeding after vascular damage by participating in hemostasis, wound healing, and thrombosis. In addition, increasing attention is focused on platelets’ role in immune regulation and the inflammatory response [2]. Additionally, platelets may also serve as indicators of various diseases, such as cardiovascular conditions [3]. Activation, as well as platelet adhesion and aggregation, can be induced by agonists such as ADP, thrombin and collagen. Due to activation, platelets secrete various substances that affect both physiological and pathophysiological processes [1]. It has been suggested that increased platelet activity and the resulting platelet hyperreactivity may be associated with an increased risk of thrombosis, as well as adverse cardiovascular events. Platelet hyperreactivity is an individual characteristic, but the mechanisms responsible for increased platelet reactivity in individuals are still not sufficiently investigated [4].

A number of antiplatelet drugs are currently available, including inhibitors of cyclooxygenase-1 (COX-1), inhibitors of phosphodiesterases (PDEs) and prostaglandin I2 (PGI2) signaling pathways, antagonists of membrane receptors such as P2Y12 receptors and PAR1 antagonists, and inhibitors of platelet aggregation such as glycoprotein IIb/IIIa (GPIIb/IIIa) antagonists [5]. An important part of the assessment of the effects of new-generation antiplatelet drugs [6,7] is to pay close attention to the inhibition of platelet glycoprotein VI (GPVI) [8]. GPVI is a glycoprotein specific to platelets that plays a crucial role in both platelet aggregation and activation induced by collagen. Under physiological conditions, when a vessel is ruptured, platelets interact with collagen via the GPVI receptor. This leads to platelet activation and adhesion, which are necessary processes for clot formation and inhibition of bleeding in the damaged vessel [9,10]. GPVI is being investigated for new antiplatelet drugs due to the fact that treatment with GPVI inhibitors would not affect hemostasis and would not induce an increased risk of hemorrhagic complications with long-term antiplatelet therapy [9,11].

Cell membranes are composed of phospholipids, which are released from the membrane using phospholipase A2 (PLA2). One of the components of phospholipids is omega-3 polyunsaturated fatty acids (omega-3 PUFAs). Omega-3 PUFAs are precursors for substances such as thromboxanes (TX), prostaglandins (PG), or leukotrienes (LT), which are known as eicosanoids and have a variety of biological properties [12,13,14,15]. Omega-3 PUFAs, found mainly in fish, have pleiotropic properties. The beneficial effects of omega-3 PUFAs on human health are well-known but still controversial [16,17]. Scientific evidence points to the cardioprotective effects of omega-3 PUFAs supplementation in patients with cardiovascular disease (CVD) and in those with increased cardiovascular risk [18,19]. Analyzing the effects of omega-3 PUFAs on hemostasis, studies on platelets have received the most attention [18]. One of the causes of CVD may be increased platelet aggregation and platelet hyperreactivity [3,20]. The theory of cellular regulation of platelet activation suggests that platelet reactivity plays a significant role in CVD pathophysiology [3]. It is suggested that one of the crucial cardioprotective mechanisms involves precisely omega-3 PUFAs, which are regarded as inhibitors of platelet reactivity (collagen and ADP receptors) [19,21]. There is still a lack of understanding of the precise mechanisms by which omega-3 PUFAs affect the platelets, so their precise effects on patient outcomes have not been fully clarified [21,22,23].

Among the pro-resolving mediators, otherwise known as specialized pro-resolving mediators (SPMs), resolvins should be emphasized, due to their anti-inflammatory properties. Resolvins are produced from both eicosapentaenoic (EPA) and docosahexaenoic acid (DHA)—resolvin E and resolvin D, respectively [24,25]. EPA has anti-inflammatory effects [13] and is a substrate for the production of TXA3 in platelets, which has a minor pro-aggregative effect, and PGI3 in the endothelium, which has anti-aggregative properties [12]. Being incorporated into the cell membrane, EPA regulates hemostasis and thrombin generation. Due to its anticoagulant and antiplatelet effects, it contributes to the reduction of adverse cardiovascular events [12,26,27,28]. In addition, it leads to the inhibiting of thromboxane-induced platelet aggregation and reducing the acute inflammatory response [12,29]. A study by Phang et al. showed that EPA reduced collagen-induced platelet aggregation in both men and women [30]. Also, a meta-analysis, involving 15 randomized controlled trials, by Gao et al. confirmed the antiplatelet properties of omega-3 PUFAs by noting the inhibition of platelet aggregation [31]. In a study done in vivo, Park et al. reported that EPA reduced platelet activation, thereby potentially reducing platelet aggregation [32].

The mechanism of this action is explained as follows: if EPA is present, competes with arachidonic acid (AA) for the enzyme present in platelets, COX, which converts EPA to TXA3 (weak pro-aggregative effect) and AA to TXA2 (pro-aggregative effect), respectively. In turn, in the vessel wall, EPA is a precursor for PGI3 with anti-aggregative properties, whereas AA is a precursor for PGI2 with also anti-aggregative properties [33]. Both synthetic and model membrane studies have confirmed that EPA, through its ability to rapidly incorporate into the membrane, changes its composition. In turn, this results in the modification of membrane properties [34,35]. Also, supplementation with omega-3 PUFAs may contribute to this effect. Additionally, EPA is responsible for reducing membrane fluidity as well as cholesterol domain formation and facilitates direct interaction between cellular components and atherosclerotic plaques, which is crucial to atherosclerosis. Due to its lipophilicity, it directly intercalates into lipoproteins and lipid bilayers. This allows it to participate in platelet activation and inflammation [34].

In light of the pleiotropic nature of omega-3 PUFAs, we considered whether the EPA metabolite—resolvin E1 had anti-aggregative properties. We hypothesized that resolvin E1 decreases platelet activation and reactivity. The effect of resolvin E1 on platelets via the collagen receptors is extremely poorly reported in the literature [12], so a novel approach was to use collagen (instead of more common agonists such as ADP) to verify the effect of resolvin E1 on platelets via the platelet receptor for collagen.

## 2. Results

### 2.1. Characteristics of Participants

Fifty women (53%) and forty-five men (47%) with an average age of 40 ± 15 years participated in the study. The basic characteristics of some morphology parameters are shown in Table 1. All other morphological and biochemical parameters are shown in the Supplementary Materials (Table S1). The participants in the study did not use antiplatelet or anticoagulant drugs and were not diagnosed with inflammatory, or chronic diseases such as diabetes, hypertension, or dyslipidemia.

### 2.2. Effect of Resolvin E1 on Platelet Aggregation in Whole Blood

To assess if resolvin E1 affects platelet reactivity in whole blood, collagen-induced platelet aggregation (2 µg/mL) was performed using two concentrations of the analyzed compound—10 nM and 100 nM. Measurements were carried out via impedance aggregometry (*n* = 10). No statistically significant differences were observed in the effect of resolvin E1 on platelet aggregation in whole blood in the presence of EPA’s metabolite at both concentrations (Table 2).

### 2.3. Effect of Resolvin E1 on Platelet Aggregation in Platelet-Rich Plasma

**Figure 1:**
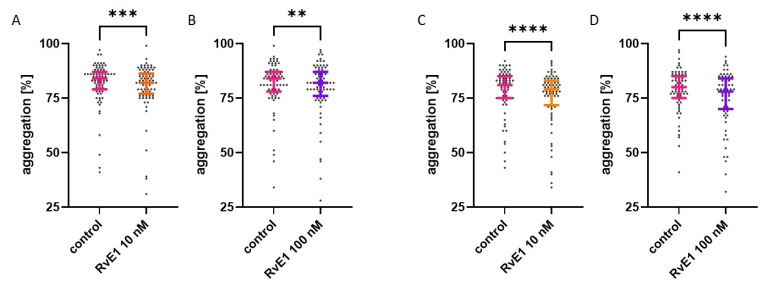
Effect of resolvin E1 (RvE1) on collagen-induced platelet aggregation in platelet-rich plasma ((**A**,**B**)—platelets at rest (*n* = 80); (**C**,**D**)—platelets after 1 h at RT (*n* = 74)). The graphs present the dot-plots with median (horizontal bar) and IQR (whiskers). The significance of differences was assessed by Wilcoxon’s signed-rank test. ** *p* < 0.01; *** *p* < 0.001; **** *p* < 0.0001.

Platelet reactivity in the presence of resolvin E1 at two concentrations (10 nM and 100 nM) was assessed by optical aggregation measurements in platelet-rich plasma. Collagen-induced platelet aggregation (2 µg/mL) was measured in paired assays (control vs. 10 nM resolvin E1 or control vs. 100 nM resolvin E1) for platelets at rest (*n* = 80) and platelets after 1 h at room temperature (RT) (*n* = 74). Platelet aggregation at rest was statistically significantly lower for both concentrations compared to control and were respectively: 82.0% (77.0–86.3) vs. 84.0% (79.0–87.0) for 10 nM resolvin E1 (*p* < 0.001) and 82.0% (76.0–87.0) vs. 84.0% (78.0–87.0) for 100 nM resolvin E1 (*p* < 0.01). Significant differences were also noted after 60 min at RT for both resolvin E1 concentrations compared to control: 10 nM resolvin E1 (*p* < 0.0001)—79.0% (71.8–83.0) vs. 81.0% (75.0–85.0) and 100 nM resolvin E1 (*p* < 0.0001)—78.0% (70.0–84.0) vs. 80.0% (75.0–85.0). Data showing the maximum aggregation value were presented in Figure 1. Statistically significant differences were also noted for another parameter describing platelet aggregation—the area under the curve (AUC). The results were presented in the Supplementary Materials (Table S2). These results suggest that resolvin E1 at concentrations of 10 nM and 100 nM may reduce platelet reactivity by inhibiting collagen-induced platelet aggregation.

### 2.4. Effect of Resolvin E1 on Platelet Aggregation in Isolated Platelets

**Figure 2:**
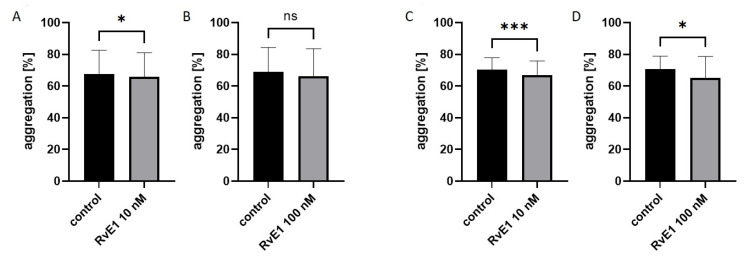
Effect of resolvin E1 (RvE1) on collagen-induced platelet aggregation in isolated platelets (*n* = 10). ((**A**,**B**)—platelets at rest; (**C**,**D**)—platelets after 1 h at RT). Data were presented as mean ± SD. The significance of differences was assessed by the paired Student’s *t*-test, ns: not significant; * *p* < 0.05; *** *p* < 0.001.

Collagen-induced optical aggregation (2 µg/mL) was also performed on isolated platelets (*n* = 10). Platelet reactivity was evaluated in paired assays after the addition of resolvin E1 at concentrations of 10 nM and 100 nM to isolated platelets. Platelet aggregation at rest was statistically significantly lower only for 10 nM resolvin E1 (*p* < 0.05) compared to controls: 65.9 ± 15.1 vs. 67.7 ± 14.9%. While, after 60 min at RT significant differences were noted for both concentrations, 10 nM resolvin E1 (*p* < 0.001) and 100 nM resolvin E1 (*p* < 0.05) compare to control, and were respectively: 67.0 ± 8.9% vs. 70.4 ± 7.5% and 65.1 ± 13.5% vs. 70.7 ± 8.2%. Data showing the maximum aggregation value were presented in Figure 2. Statistically significant differences were also found for the second parameter describing platelet aggregation—AUC. The results were presented in the Supplementary Materials (Table S3). The results obtained suggest that resolvin E1 at concentrations of 10 nM and 100 nM may reduce platelet reactivity by inhibiting collagen-induced platelet aggregation.

### 2.5. Effect of Resolvin E1 on P-Selectin (CD62) Expression in Platelet-Rich Plasma

Platelet-rich plasma incubated with two concentrations of resolvin E1 (10 nM and 100 nM) was used to measure the expression of P-selectin, taking into account the sample without activation and with collagen (10 µg/mL) activation after 5 min. No statistically significant differences were observed in resolvin E1’s effect on platelet activation (no agonist) independently on the time incubation (at rest or after 1 h). However, while assessing the fraction of CD62-positive platelets after activation with collagen in the presence of resolvin E1, the statistical significance was shown for both resolvin E1 concentrations (10 nM and 100 nM) only for platelets at rest, with a decreasing tendency in the amount of CD62-positive platelets after 1 h at RT (Table 3).

### 2.6. Effect of Resolvin E1 on the Fibrinogen-Binding Capacity to Platelets in Platelet-Rich Plasma

Platelet activation and reactivity (10 µg/mL collagen activation) was also assessed based on measurements of the exogenous fibrinogen-binding capacity to platelets in platelet-rich plasma in the presence of resolvin E1 at concentrations of 10 nM and 100 nM. A decreasing tendency was observed in the number of fibrinogen-positive platelets, but there were no statistically significant differences in the effects of resolvin E1 on both platelet activation (except 100 nM resolvin E1 in platelets after 1 h at RT, *p* = 0.040) and reactivity (Table 4).

### 2.7. Effect of Resolvin E1 on Platelet Membrane Fluidity in Isolated Platelets

**Figure 3:**
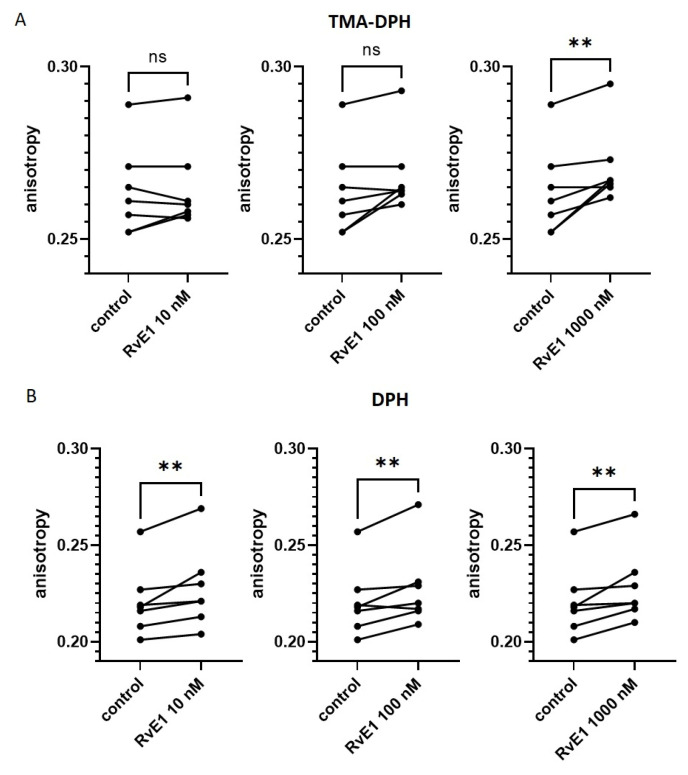
Effect of resolvin E1 (RvE1) on platelet membrane fluidity with fluorescent probes: (**A**) TMA-DPH (*n* = 7) and (**B**) DPH (*n* = 7). The significance of differences was assessed by the Friedman test with Dunn’s multiple comparisons test (TMA-DPH) and by the ANOVA with Dunnett’s multiple comparisons test (DPH), ns: not significant; ** *p* < 0.01.

Platelet membrane fluidity after incubation with resolvin E1 at concentrations of 10 nM, 100 nM and 1000 nM, followed by incubation with fluorescent probes (TMA-DPH and DPH), was analyzed by measuring fluorescence anisotropy using a spectrofluorometer (*n* = 7). A slight increase in fluorescence anisotropy (both TMA-DPH and DPH) was observed in the presence of resolvin E1 (concentration-dependent). Results were presented in Figure 3.

## 3. Discussion

The pleiotropic effects of omega-3 PUFAs based on their anti-aggregative, anti-platelet, and anti-inflammatory properties, and the general importance of omega-3 PUFAs, especially EPA, in supporting traditional therapy by regulating platelet function should not be questioned [21].

In our study, we investigated the effect of resolvin E1 on collagen-induced platelet aggregation in three environments: isolated platelets, platelet-rich plasma, and whole blood. However, the anti-aggregative potential of the EPA’s metabolite on platelets was noted only in platelet-rich plasma and in isolated platelets—statistically significant results for both parameters of platelet reactivity (maximal platelet aggregation and area under the aggregation curve). Analyzing the results of collagen-induced platelet aggregation in whole blood in the presence of resolvin E1, we only noticed a decreasing tendency for the concentration of 100 nM of resolvin E1 compared to control, both for platelets at rest and after 1 h at RT (statistically non-significant data). We also evaluated the effect of resolvin E1 on platelet activation and reactivity by flow cytometry. Platelet-rich plasma was used to measure P-selectin exposure on platelets and the exogenous fibrinogen-binding capacity of platelets. The flow cytometry method, which detects antigens present inside the cells or on the cell surface, allows the analysis of platelet activation processes. This determines the degree of intracellular activation and characterizes the platelets’ state before extravasation. In turn, reactivity is assessed using platelet activation parameters after induction. In our work, we have demonstrated that resolvin E1 does not affect platelet activation in vitro and collagen-induced platelet activation (reactivity) based on exogenous fibrinogen binding to platelets. However, we noted that resolvin E1 at both concentrations (10 nM and 100 nM) statistically significantly decreases platelet reactivity (after collagen activation) based on P-selectin expression, but only in platelets at rest. 

A study by Dona et al. showed that resolvin E1 was responsible for the rapid regulation of leukocyte expression of adhesion molecules (study using human whole blood) [29]. On the other hand, in the study by Fredman et al. resolvin E1 inhibited platelet aggregation induced by ADP and thromboxane receptor agonist (U46619)—the effect was concentration-dependent (studies using platelet-rich plasma) [36]. In contrast, in the aforementioned study by Dona et al., no blocking effect of resolvin E1 on collagen-induced platelet aggregation was observed. The discrepancies obtained from the results from our study and those from Dona et al. may result from differences in the number of aggregation measurements performed in platelet-rich plasma, as well as the concentration and type of agonist used. In our study, we carried out significantly more measurements (*n* = 80 for platelets at rest and *n* = 74 for platelets after 1 h at RT) and used freshly opened collagen (with a concentration of 2 µg/mL), characterized by lower activity, which may have contributed to the noticeable effect of resolvin E1 on platelet aggregation in platelet-rich plasma. In contrast, in the study by Dona et al. only 4 aggregation measurements were performed, with collagen at a concentration of 1.5 µg/mL. Unfortunately, no more detailed information was given on the agonist that was used, but it is worth noting that collagen undergoes polymerization during prolonged storage, thus increasing its activity. It is also worth mentioning that possible differences between aggregation in whole blood and platelet-rich plasma may result from the presence in whole blood also of leukocytes and erythrocytes, which are absent in platelet-rich plasma. It is obvious that whole blood reflects the physiological condition, and thus allows to reflect the in vivo effects. However, considering the potential mechanism regarding the effect of resolvin E1 on platelets by affecting the structure of the platelet cell membrane, the blood morphotic elements present in whole blood (in addition to platelets) may influence aggregation. Resolvin E1 may interact with other cells, also incorporating into the membrane of erythrocytes, for example, which may result in a reduction in its available amount for platelets and may suggest the need for using higher concentrations of resolvin E1 for aggregation in whole blood. Obviously, this hypothesis needs to be confirmed in further studies. A study by Fredman et al. reported decreased P-selectin expression after ADP stimulation in platelets incubated with resolvin at concentrations of 0.1–100 nM. Noteworthy, the platelet receptor ChemR23 is required for the effect to occur. Resolvin E1 may specifically impact platelets by activating platelets through ADP, suggesting a novel cellular mechanism. EPA may not only affect the reduction of vascular inflammation but also ADP-dependent platelet activation, which is extremely significant in the pathogenesis of CVD and the occurrence of adverse cardiovascular events [36]. Undoubtedly, resolvin E1 has protective properties against cells, especially platelets [36]. However, despite this, there is still insufficient evidence for the anticoagulant properties of omega-3 PUFAs to explain precisely the action at the cellular/molecular level, as highlighted in the study by Stupin et al., considering resolvin E1 as a specialized proinflammatory mediator that is produced in response to the resolution of acute inflammation [37]. A reduction in platelet activation and maintenance of platelet function even after one week of cold storage was reported after treatment with resolvin E1 in a study by Reddoch-Cardenas et al. [38]. 

We also assessed the effect of resolvin E1 on platelet membrane fluidity and observed a slight increase in fluorescence anisotropy (both TMA-DPH and DPH), which may indicate maintenance of platelet membrane structure (without increasing fluidity) in the presence of resolvin E1 (concentration-dependent). According to the literature, EPA maintains the membrane structure without altering its fluidity or promoting the cholesterol domain formation. In turn, DHA increases cell membrane fluidity and affects lipid modification [34]. There are a few studies indicating that EPA increases membrane fluidity [39,40], however, there are definitely more data underlining the stabilizing influence of EPA on the cell membrane [34,41,42,43]. Differences in EPA and DHA effects on cell membrane structure may also indicate their different effects on humans. It has been suggested that EPA has an increased potential for cardiovascular events [34].

In general, over-the-counter omega-3 PUFAs preparations are not recommended for reducing cardiovascular risk in both patients with and without CVD. However, attention should be given to preparations available by prescription, such as the EPA derivative, ethyl ester—icosapent ethyl (IPE). In the REDUCE-IT study (Reduction of Cardiovascular Events with Icosapent Ethyl–Intervention Trial) [44] triglyceride levels and adverse cardiovascular event rates were both significantly reduced after administration of IPE. In contrast, other studies in which patients received products containing EPA and DHA together, showed no significant reduction in cardiovascular events among high-risk patients [45,46]. The efficacy of this effect can be closely associated with the purified EPA ethyl ester product, so our research has focused on EPA and its metabolites. Studies have shown that IPE can have a multi-faceted effect, affecting not only triglyceride levels, but also inflammation and platelet functions. Clarifying the exact mechanisms would undeniably support interventions and the reduction of cardiovascular mortality, which is still one of the leading causes of death worldwide [47].

A recent study by Back et al. highlighted resolvin E1, which is an EPA-derived lipid mediator that acts through the ChemR23 receptor and contributes to the reduction of inflammation associated with other CVD conditions. The role of the EPA-resolvin E1-ChemR23 axis was pointed out as a key mechanism leading to a reduction in inflammation and thus a positive impact on cardiovascular event prevention [48]. 

In contrast, the effect of resolvin E1 on platelets via the collagen receptor is extremely poorly reported in the literature [49]. As part of our research, we focused on this mechanism, which can be considered a novel approach to the project. A receptor on platelets that plays a crucial role in vessel disruption is GPVI. It attaches to collagen in the endothelium at the site of plaque rupture and binds platelets there. As a result of this process, platelet activation and aggregation occur to form a stable vascular plug. Many studies including epidemiological and preclinical experiments have used mouse models with a peptide or genetic deficiency of GPVI. The results of these studies have confirmed that this receptor is not only effective but equally a safe antiplatelet target. Despite this demonstration, few drugs have been identified that act antagonistically against GPVI (Revacept, Glenzocimab) [50,51].

Bearing in mind the controversial results of omega-3 PUFAs supplementation [52] our report is a voice in the discussion on the use of omega-3 PUFAs metabolites. In our study, we mainly used the following concentrations of resolvin: 10 nM and 100 nM, which were chosen based on the available scientific literature. In vitro studies performed by other researchers mostly used resolvin E1 concentrations in the range of 1.0 nM–1.0 µM [29]. In a study by Al-Shaer et al. [53] obese adults consumed marine oil in a total dose of 2 g/day for 1 month. The aim of this clinical trial was to verify if this supplementation would result in an increase of SPM metabolites in plasma, one of which is resolvin E1. A statistically significant (*p* = 0.008) increase of up to 3.5-fold in the plasma of EPA metabolites, including resolvin E1, was noted. This interventional study showed that after one month of marine oil supplementation, plasma levels of resolvin E1 can range from 1 to 5 ng/mL [53]. The concentration of resolvin E1 used in our study—10 nM—after appropriate recalculation is within the range mentioned above, so it may be achievable after proper supplementation with omega-3 fatty acids or their metabolites. Thus, we conclude that resolvin E1 at a concentration of 10 nM deserves further research. Therefore, in our work, we indicate an innovative research direction, concentrating on further studies leading to the use of omega-3 PUFAs metabolite-based preparations instead of traditional omega-3 PUFAs supplementation. A combination of natural products’ health-promoting properties with the efficacy and safety of antiplatelet drugs may be crucial in modern antiplatelet therapy. We investigated that resolvin E1 may inhibit platelet reactivity due to the reduction of collagen-induced platelet aggregation in platelet-rich plasma and isolated platelets. In conclusion, this was the first attempt to explore and clarify the molecular mechanism of resolvin E1’s impact on platelets, aimed at connecting platelet membrane structure maintenance with the anti-aggregative effect of this compound. The association between platelet reactivity and platelet cell membrane fluidity requires further research. Therefore, there is undoubtedly a need to determine if resolvin E1 is also an inhibitor of GPVI. In addition, it is necessary to prove the synergistic effects of resolvin E1 and GPVI on platelet reactivity. The results of our study suggest that resolvin E1, a metabolite of omega-3 PUFAs, has anti-platelet and anti-aggregative potential. This study may provide the basis for a prospective randomized intervention study to use EPA and resolvin E1 as crucial components in the prevention of civilization diseases (in particular CVD) in the future, since they can reduce platelet reactivity and therefore increase the efficacy of antiplatelet drugs. 

## 4. Materials and Methods

### 4.1. Chemicals

Antibodies for flow cytometry (anti-human CD61/FITC, CD61/PE, CD62/PE), Cellfix, and 0.105 M buffered sodium citrate or acid-citrate-dextrose (ACD) were obtained from Becton-Dickinson (Franklin Lakes, NJ, USA). Bovine serum albumin (BSA), tetrahydrofuran (THF), trimethylammonium diphenylhexatriene (TMA-DPH), diphenylhexatriene (DPH), and prostaglandin E1 were purchased from Sigma (St. Louis, MO, USA). Phosphate buffered saline (PBS) was provided from Corning (New York, NY, USA). Fibrinogen from Human Plasma Oregon Green 488 Conjugate was purchased from Invitrogen (Carlsbad, CA, USA). Resolvin E1 was from Cayman Chemical (Ann Arbor, MI, USA). Collagen was obtained from Chrono-log Co (Havertown, PA, USA). All other chemicals, unless otherwise stated, were supplied by Avantor Performance Materials Poland S.A. (Gliwice, Poland).

### 4.2. Study Population—Recruitment

Ninety-five people aged 20 to 70 participated in this study. Healthy volunteers were recruited at the Department of Haemostasis and Haemostatic Disorders (Medical University of Lodz) between March 2021 and November 2022, according to the inclusion and exclusion criteria. Donors were excluded from the study if they were taking antiplatelet and anticoagulant medicaments or other drugs affecting blood clotting for at least two weeks before the study. Participants were also not included in the study if they were infected during recruitment, had a C-reactive protein level higher than 10 mg/mL, had cancer diagnosis, or had chronic inflammatory disease. All the participants were introduced to the aim of the study and enrolled after giving their informed, voluntary, and written consent. The research was carried out following approval by the Bioethical Commission at the Medical University of Lodz on June 2020 (RNN/153/20/KE). 

### 4.3. Blood Collection and Preparation

Blood from healthy donors was collected in a single donation, fasting in the morning between 8 a.m. and 9 a.m. into polypropylene tubes (S-Monovette^®^) with a total volume of no more than 30 mL. The tubes for assays with whole blood and platelet-rich plasma (PRP) contained a 3.2% (0.105 M) buffered sodium citrate solution (with blood to anticoagulant volume ratio of 9:1). The tubes for assays with isolated platelets included acid citrate dextrose (ACD, with blood to anticoagulant volume ratio of 6:1). Immediately after collection, the blood tubes were gently inverted to allow the anticoagulant to mix equally with the blood. For further experiments, the whole blood was centrifuged at 190× *g* for 12 min at 37 °C to obtain PRP. The remaining whole blood fraction after PRP removal was centrifuged again at 800× *g* for 15 min at 37 °C to obtain platelet-poor plasma (PPP). Whole blood and then PRP for platelet isolation contained prostaglandin E1 (at 50 ng/mL). The platelet suspension was obtained by centrifuging the PRP at 800× *g* for 15 min at 37 °C, and then the sediment was resuspended in Tyrode’s buffer (134 mM NaCl, 12 mM NaHCO_3_, 2.9 mM KCl, 0.34 mM Na_2_HPO_4_, 1 mM MgCl_2_, 10 mM HEPES, 5 mM glucose, pH 7.4) containing 0.2% bovine serum albumin (BSA). Platelet counts were determined using an automatic hematology analyzer Sysmex XS-800i™ (Sysmex, Kobe, Japan) and then diluted using Tyrode’s buffer to 2 × 10^8^ cells/mL for optical aggregation, P-selectin expression, and fibrinogen-binding, and to 1 × 10^6^ cells/mL for platelet membrane fluidity. 

### 4.4. Parameters from Laboratory Results

Each donor received (in cooperation with the hospital laboratory at Medical University of Lodz) a wide panel of laboratory tests, including complete blood morphology, and biochemical parameters (e.g., C-reactive protein, glucose, lipidogram).

### 4.5. Chemical Formula of Eicosapentaenoic Acid (EPA) and Its Metabolite (Resolvin E1)

**Figure 4:**
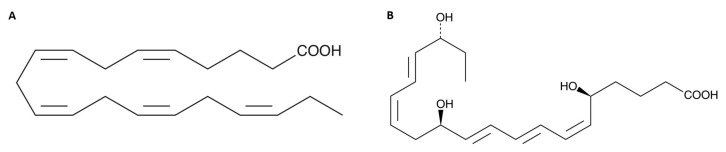
Chemical formula of (**A**) eicosapentaenoic acid (EPA) and (**B**) resolvin E1—EPA’s metabolite. Data taken from the Cayman Chemical’s website (Item No. 90110 and Item No. 10007848, respectively).

The compound used in this study was resolvin E1, which is a metabolite of eicosapentaenoic acid (EPA), a member of the omega-3 polyunsaturated fatty acids (omega-3 PUFAs). In Figure 4, the chemical formulas of EPA and resolvin E1 are shown.

### 4.6. Platelet Aggregation in Whole Blood

Whole blood collected from healthy donors (*n* = 10) was diluted with 300 µL 0.9% NaCl (with a blood-to-saline volume ratio of 1:1) and rested at 37 °C for 3 min. Then samples were incubated with 0.25% ethanol (control) and two concentrations of resolvin E1 (10 nM and 100 nM) at 37 °C for 10 min, following which platelet aggregation was induced with collagen (2 µg/mL). Aggregation was recorded both at rest (0–10 min) and after 60 min at RT. All the samples were measured using a Multiplate analyzer impedance aggregometer (Roche, Basel, Switzerland) for 6 min. Results of the area under the curve (AUC) were presented in arbitrary units (U) as median and quartile ranges.

### 4.7. Platelet Aggregation in Platelet-Rich Plasma

This part of the experiment was designed to evaluate platelet reactivity with a particular reference to the effect of resolvin E1 on collagen-induced platelet aggregation. Whole blood collected from healthy donors was centrifuged to obtain PRP (190× *g*, 37 °C for 12 min) and then into PPP (800× *g*, 37 °C for 15 min), which was used as a blank. The prepared PRP samples with a final concentration of 200 × 10^6^ platelets/mL were incubated with 0.25% ethanol as a control and two concentrations of resolvin E1 (10 nM and 100 nM), at 37 °C for 10 min. Then platelet aggregation was induced with collagen (2 µg/mL) and was carried out for 10 min. Measurements were performed in paired assays. Aggregation was recorded both at rest (0–10 min) and after 60 min at RT. All samples were measured using an optical aggregometer (Chrono-Log 490-4D, Havertown, PA, USA). The results were presented as the maximum aggregation value (A_max_) expressed in %. 

### 4.8. Platelet Aggregation in Isolated Platelets

The principle of this method is the same as optical aggregation with PRP described above. Whole blood was centrifuged at 37 °C and 190× *g* for 12 min with prostaglandin E1 (50 ng/mL) to prevent excessive platelet activation. The resulting PRP was centrifuged again for 15 min at 37 °C and 700× *g*. Measurements were performed in paired assays, both at rest (0–10 min) and after 60 min at RT, and with Tyrode’s buffer as a blank. Samples were incubated with 0.25% ethanol (control sample) and two concentrations of resolvin E1 (10 nM and 100 nM), at 37 °C for 10 min. Next, platelet aggregation in response to 2 µg/mL collagen was monitored for 10 min in suspensions of isolated platelets with a platelet count of 200 × 10^6^ platelets/mL using an optical aggregometer (Chrono-Log 490-4D, Havertown, PA, USA). The results were presented as the maximum aggregation value (A_max_) expressed in %. 

### 4.9. Platelet Activation and Reactivity 

To evaluate the effect of resolvin E1 on platelet activation and reactivity, the following antibodies were used: anti-CD61 (PE-labeled), anti-CD62 (PE-labeled), and anti-CD61 (FITC-labeled). PRP was used to measure P-selectin (CD62) expression on platelets and the fibrinogen-binding capacity to platelets, considering the samples without activation and with activation with collagen (5 min). The experiments in both cases included a control sample (with ethanol), with 10 nM and 100 nM resolvin E1. The measurement was performed at rest (0–10 min) and after 60 min at RT incubation. 

#### 4.9.1. P-Selectin Expression

PRP was initially preincubated with 10 nM and 100 nM resolvin E1 for 10 min at 37 °C and then was activated with 10 µg/mL collagen for 5 min at RT, after which the samples were labeled with the appropriate antibodies for 15 min at RT (anti-CD61/FITC for platelet gating and anti-CD62/PE for detection of activated platelets), and then samples were fixed with CellFix for 1 h at RT or for 30 min at 37 °C. Before measuring, samples were diluted in phosphate-buffered saline (PBS). The measurement of the fraction of activated platelets, exactly the surface expression of CD62P (P-selectin) in platelets, was performed using a FACSCanto II flow cytometer (Becton-Dickinson, Franklin Lakes, NJ, USA). 10,000 cells (CD61/FITC-positive subjects) were collected, and results were presented as a percentage of marker-positive platelets.

#### 4.9.2. Fibrinogen-Binding

PRP was initially preincubated with 10 nM and 100 nM resolvin E1 for 10 min at 37 °C. Exogenous fibrinogen (labeled with 3 µg/mL Oregon Green) was then added to the samples and activated with 10 µg/mL collagen for 5 min at RT. After that samples were diluted with PBS, labeled with the appropriate antibodies for 15 min at RT (anti-CD61/PE for platelet gating), and then fixed with CellFix for 1 h at RT or for 30 min at 37 °C. Before measuring, samples were diluted in PBS. Due to platelet activation, there is a change in the conformation of the glycoprotein IIb/IIIa complex. This exposes the fibrinogen-binding site and enables fibrinogen-binding by platelets. The measurement of the fraction of activated platelets was performed using a FACSCanto II flow cytometer (Becton-Dickinson, Franklin Lakes, NJ, USA). 10,000 cells (CD61/PE-positive subjects) were collected, and results were presented as a percentage of marker-positive platelets.

### 4.10. Platelet Membrane Fluidity

Platelet membrane fluidity was assessed based on the measurement of fluorescence anisotropy using a PerkinElmer LS-50B spectrofluorometer (Waltham, MA, USA). Isolated platelets were incubated at 37 °C for 10 min with 0.25% ethanol (control) and resolvin E1 at three concentrations: 10 nM, 100 nM and 1000 nM. Afterward, platelets were incubated at 37 °C in the dark with two probes dissolved in tetrahydrofuran (THF): 1.5 µM TMA-DPH (trimethylammonium diphenylhexatriene) for 5 min or 1.5 µM DPH (diphenylhexatriene) for 45 min. Measurements were performed at λ_ex_ = 358 nm and λ_em_ = 428 nm for TMA-DPH and at λ_ex_ = 348 nm and λ_em_ = 426 nm for DPH, measuring I_v_ (intensity of vertically polarized light) and I_h_ (intensity of horizontally polarized light) at G = 1.4 (a correction factor for the r value). The slit width was 15 nm (the excitation monochromator) and 10 nm (the emission monochromator) for both probes. The anisotropy of fluorescence was calculated using the formula:

(1) $$r=\frac{IvG-Ih}{Iv+2\;Ih}$$

### 4.11. Statistical Analysis

Statistical analysis was performed using Statistica v.13 (Dell Inc., Tulsa, OH, USA) and GraphPad Prism v.9.1.1. (San Diego, CA, USA). The distribution of the analyzed variables was assessed by the Shapiro-Wilk test. Data were presented as mean ± SD (variables with normal distribution) or as median and interquartile range (IQR) (variables with non-normal distribution). Statistical significance of differences between the two groups was assessed using the paired Student’s *t*-test or the unpaired Student’s *t*-test (variables with normal distribution) and the Wilcoxon signed-rank test or the Mann-Whitney U-test (variables with non-normal distribution), while to compare differences between more than two groups, ANOVA for repeated measures with the post hoc Dunnett’s test (variables with normal distribution) or Friedman test with post hoc analysis with Dunn’s multiple comparisons tests (variables with non-normal distribution) was applied. The differences in the analyzed variables were considered statistically significant if the *p* value was <0.05.

## 5. Conclusions

In conclusion, the results of our work indicate that resolvin E1, a metabolite of EPA, has anti-aggregative potential. The innovative part of our study was to verify the effect of resolvin E1 on platelets through the collagen receptor instead of more common agonists such as ADP. Moreover, this was the first attempt to explore and clarify the molecular mechanism of resolvin E1’s impact on platelets. Our report is also a voice in the discussion on the use of omega-3 PUFAs’ metabolites. We indicate an innovative research direction, concentrating on the further analysis leading to the use of omega-3 PUFAs metabolite-based preparations instead of traditional omega-3 PUFAs supplementation.

## Figures and Tables

**Table 1:** The basic characteristics of morphology parameters among healthy donors (*n* = 95).

Parameter	Healthy Donors (*n* = 95)
PLT [×10^3^/µL]	253 ± 53
MPV [fL]	10.3 (9.9–11.0)
PCT [%]	0.25 (0.23–0.29)
PDW [fL]	11.7 (10.9–13.3)
RBC [×10^6^/µL]	4.78 ± 0.48
WBC [×10^3^/µL]	6.11 ± 1.40

Data were presented as mean ± SD (normal distribution) or median (IQR) (non-normal distribution). PLT—platelets; MPV—mean platelet volume; PCT—platelet crit; PDW—platelet distribution width; RBC—red blood cells; WBC—white blood cells.

**Table 2:** Effect of resolvin E1 on collagen-induced platelet aggregation in whole blood (*n* = 10).

	Control	Resolvin E1 10 nM	*p* Value	Resolvin E1 100 nM	*p* Value
platelet at rest	59.0 (54.0–68.0)	60.0 (54.5–69.0)	0.9999	54.0 (51.0–67.5)	0.1542
platelet after 1 h at RT	51.5 (43.0–57.8)	52.0 (41.0–55.8)	0.6346	50.5 (44.3–63.0)	0.7631

Results were presented in arbitrary units (U) as median (IQR). The significance of differences was assessed by the Friedman test with Dunn’s multiple comparisons test.

**Table 3:** Effect of resolvin E1 on platelet activation and reactivity based on the expression of P-selectin (CD62) on platelets (*n* = 14).

	Control	Resolvin E1 10 nM	*p* Value	Resolvin E1 100 nM	*p* Value
platelets at rest					
no activation	1.5 (1.0–2.1)	1.4 (0.9–2.3)	0.9999	1.3 (1.1–2.3)	0.9999
collagen activation	11.9 (8.0–18.7)	10.5 (5.6–18.2)	0.0285	6.7 (5.1–17.1)	0.0012
platelets after 1 h at RT					
no activation	3.6 (1.5–5.1)	3.5 (1.8–4.6)	0.9999	3.0 (2.1–5.4)	0.9999
collagen activation	16.1 (7.6–26.1)	12.3 (7.0–24.9)	0.1324	9.5 (7.7–20.0)	0.4413

Results were presented as a percentage of CD62-positive platelets as median (IQR). The significance of differences was assessed by the Friedman test with Dunn’s multiple comparison test.

**Table 4:** Effect of resolvin E1 on platelet activation and reactivity based on exogenous fibrinogen-binding to platelets (*n* = 14).

	Control	Resolvin E1 10 nM	*p* Value	Resolvin E1 100 nM	*p* Value
platelets at rest					
no activation	3.5 ± 1.9	3.4 ± 1.8	0.8419	3.3 ± 1.6	0.6961
collagen activation	45.8 ± 18.5	44.1 ± 21.9	0.8815	43.9 ± 22.9	0.8512
platelets after 1 h at RT					
no activation	4.0 ± 3.0	3.7 ± 2.4	0.4364	3.3 ± 2.0	0.0399
collagen activation	39.0 ± 18.7	33.4 ± 20.2	0.1159	32.7 ± 20.7	0.0714

Results were presented as a percentage of fibrinogen-positive platelets as mean ± SD. The significance of differences was assessed by the ANOVA with Dunnett’s multiple comparisons test.

## Data Availability

The data presented in this study are available on request from the corresponding author. The data are not publicly available in order to protect patients privacy.

## Supplementary Materials

The following supporting information can be downloaded at: https://www.mdpi.com/article/10.3390/molecules28145323/s1, Table S1: Morphological and biochemical parameters among healthy donors. Table S2: Reactivity of collagen-induced platelets in platelet-rich plasma in the presence of resolvin E1 based on the area under the curve (AUC) parameter. Table S3: Reactivity of collagen-induced platelets in isolated platelets in the presence of resolvin E1 based on the area under the curve (AUC) parameter.
