# Supplementary material for: The Anti-Aggregative Potential of Resolvin E1 on Human Platelets
Source: Molecules. 2023 Jul 11;28(14):5323. doi: 10.3390/molecules28145323 (PMC10385542; doi:10.3390/molecules28145323)
Supplement: Supplementary file 1 [file molecules-28-05323-s001.zip › molecules-2464762-supplementary.pdf]

The Anti-Aggregative Potential of Resolvin E1 on Human Platelets

Patrycja Szymańska [1*], Bogusława Luzak [1], Katarzyna Miłowska [2], and Jacek Golański [1]

Table S1. Morphological and biochemical parameters among healthy donors.

Parameter	Healthy donors (*n* = 95)
HGB [g/dL]	14.3 (13.1-15.5)
HCT [%]	41.7 ± 4.0
MCV [fL]	87 (85-90)
MCH [pg]	30.1 (28.9-30.9)
MCHC [g/dL]	34.2 ± 0.9
RDW [%]	12.7 (12.3-13.1)
NEU [$\times 10^3/\mu L$]	3.14 (2.54-3.89)
LYM [$\times 10^3/\mu L$]	2.02 (1.70-2.40)
MON [$\times 10^3/\mu L$]	0.53 (0.46-0.62)
EOS [$\times 10^3/\mu L$]	0.16 (0.10-0.23)
BAS [$\times 10^3/\mu L$]	0.04 (0.03-0.05)
Total cholesterol [mmol/L]	5.26 ± 1.04
LDL-cholesterol [mmol/L]	3.22 ± 0.91
HDL-cholesterol [mmol/L]	1.51 (1.29-1.73)
Non-HDL-cholesterol [mmol/L]	3.73 ± 1.05
TG [mmol/dL]	1.12 (0.81-1.43)
Glucose [mmol/L]	5.25 (4.98-5.61)
Creatinine [µmol/L]	78.9 (66.8-89.6)
GFR [mL/min/1.73m^2]	96.8 ± 17.9
Uric acid [µmol/L]	319.9 ± 86.3
Bilirubin [µmol/L]	12.4 (9.0-15.7)
AST [U/L]	22.9 (19.6-26.7)
ALT [U/L]	18.9 (14.5-33.1)

Data were presented as mean ± SD (normal distribution) or median (IQR) (non-normal distribution). HGB – hemoglobin; HCT – hematocrit; MCV – mean corpuscular volume; MCH – mean corpuscular hemoglobin; MCHC – mean corpuscular hemoglobin concentration; RDW – red cell distribution width; NEU – neutrophils; LYM – lymphocytes; MON – monocytes; EOS – eosinophils; BAS – basophils; LDL – low-density lipoprotein; HDL – high-density lipoprotein; TG – triglycerides; GFR – glomerular filtration rate; AST – aspartate aminotransferase; ALT – alanine aminotransferase.

Table S2. Reactivity of collagen-induced platelets in platelet-rich plasma in the presence of resolvin E1 based on the area under the curve (AUC) parameter.

	Control	Resolvin E1 10 nM	*p* Value	Control	Resolvin E1 100 nM	*p* Value
platelet at rest	708.0 (664.0-748.3)	694.0 (649.8-732.3)	< 0.0001	705.0 (649.0-748.5)	687.5 (638.0-740.8)	0.014
platelet after 1 hour at RT	653.0 (577.3-690.3)	623.5 (551.0-672.5)	< 0.0001	637.0 (560.0-695.0)	613.0 (516.0-675.0)	< 0.0001

Results were presented as AUC (% * time) parameter as median (IQR). The significance of differences was assessed by Wilcoxon's signed-rank test.

Table S3. Reactivity of collagen-induced platelets in isolated platelets in the presence of resolvin E1 based on the area under the curve (AUC) parameter.

	**Control**	**Resolvin E1 10 nM**	***p* Value**	**Control**	**Resolvin E1 100 nM**	***p* Value**
platelet at rest	571.0 ± 137.9	554.5 ± 135.3	0.0199	572.3 ± 127.4	542.6 ± 139.8	0.0315
platelet after 1 hour at RT	568.6 ± 68.8	536.1 ± 78.2	0.0003	572.9 ± 85.9	518.7 ± 126.0	0.0053

Results were presented as AUC (% * time) parameter as mean ± SD. The significance of differences was assessed by the paired Student's t-test.
